# Supplementary figures and images for: Characterization of Dof Transcription Factors and the Heat-Tolerant Function of PeDof-11 in Passion Fruit (Passiflora edulis)
Source: Int J Mol Sci. 2023 Jul 28;24(15):12091. doi: 10.3390/ijms241512091 (PMC10418448; doi:10.3390/ijms241512091)

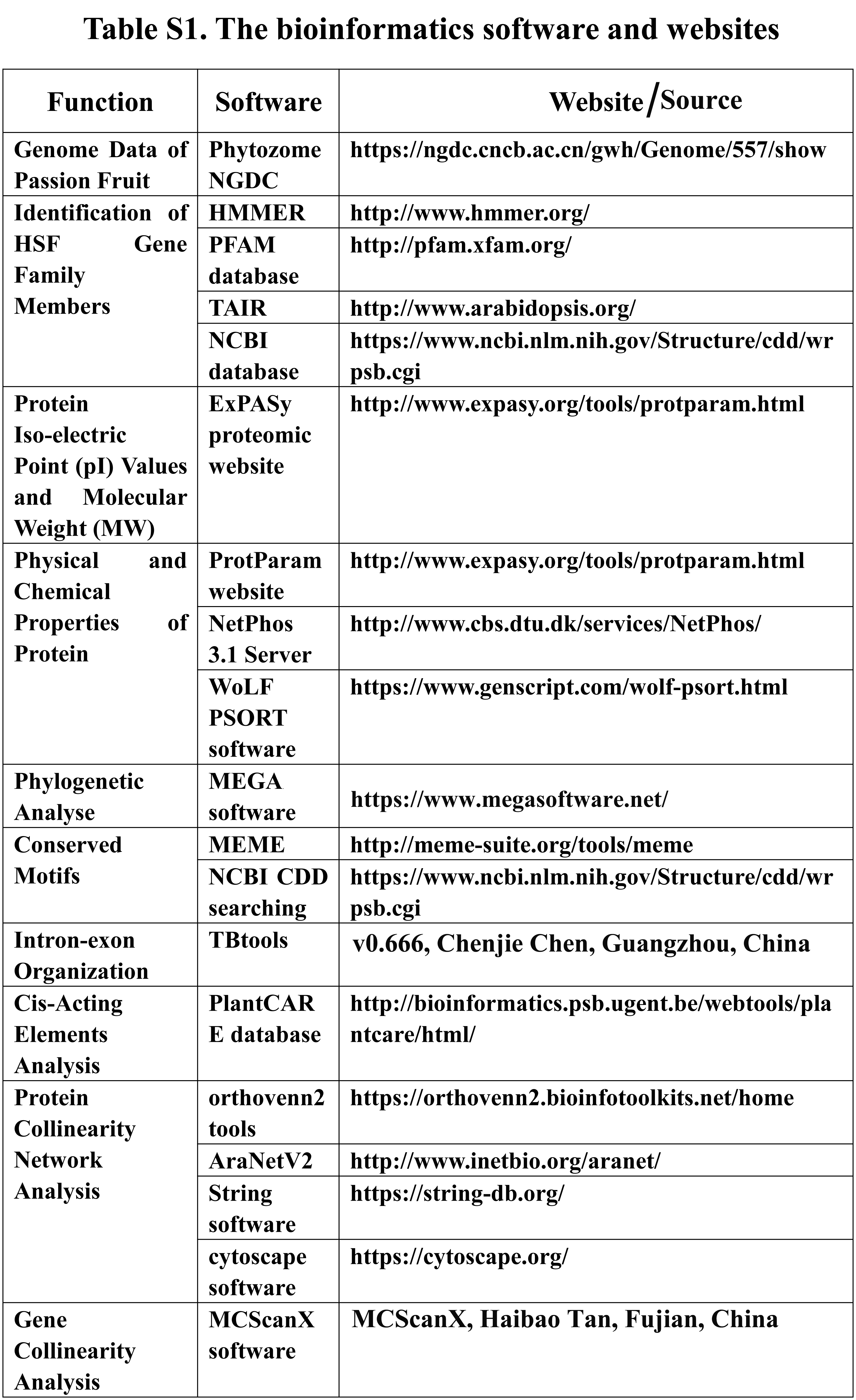

Supplement: Supplementary file 1 [file ijms-24-12091-s001.zip › Table S1 The bioinformatics software and websites.jpg]

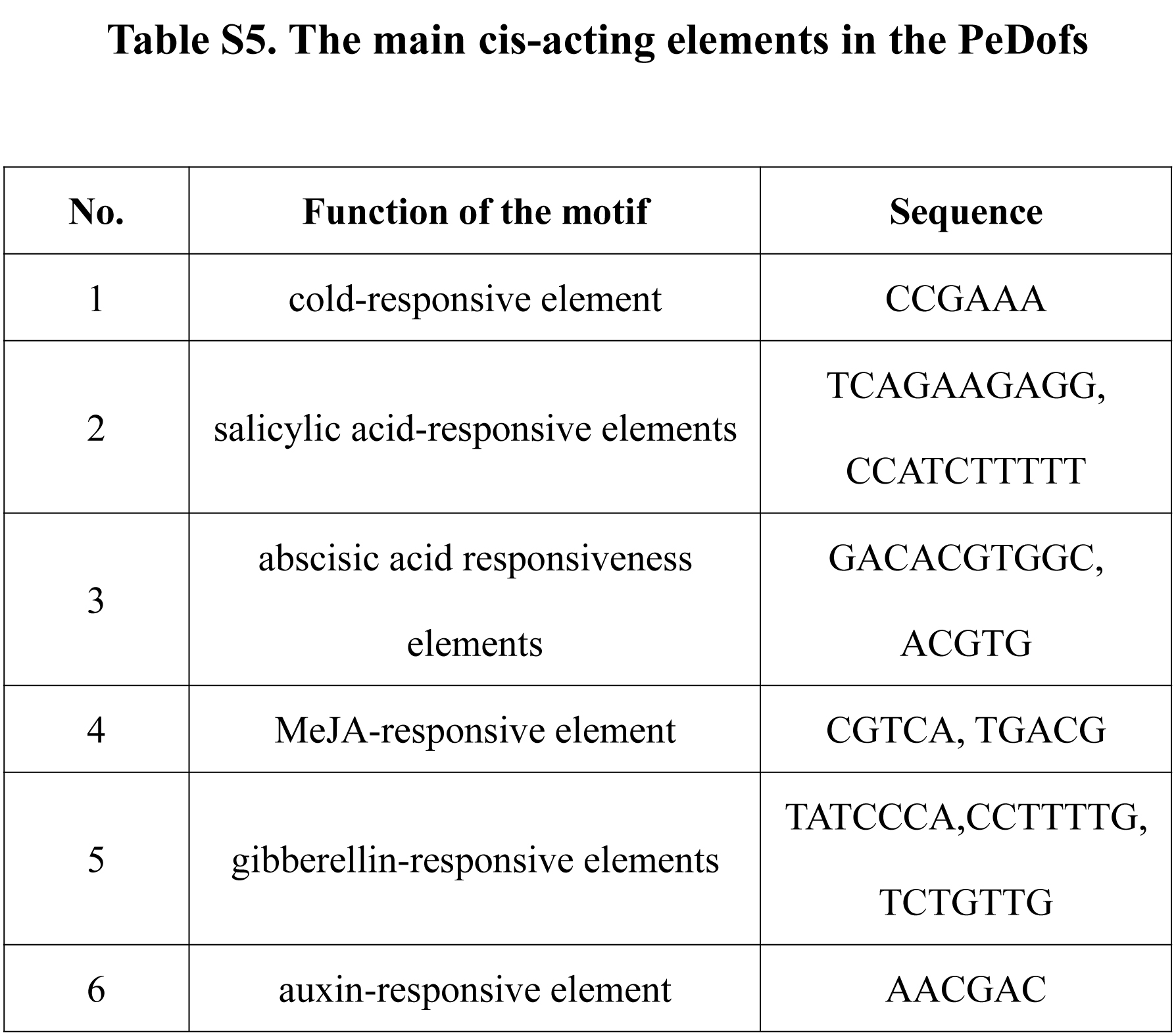

Supplement: Supplementary file 1 [file ijms-24-12091-s001.zip › Table S5 The main cis-acting elements in the PeDofs.jpg]
